# Enhancing innovation between scientific and indigenous knowledge: pioneer NGOs in India

**DOI:** 10.1186/1746-4269-5-29

**Published:** 2009-10-22

**Authors:** Maria-Costanza Torri, Julie Laplante

**Affiliations:** 1 Department of Social Sciences, University of Toronto Scarborough, 1265 Military Trail, ON M1C 1A4, Toronto, Canada; 2 Department of Sociology and Anthropology, University of Ottawa, Pavillon Desmarais, 8162 55, Laurier Est Ottawa ON K1N 6N5, Ottawa, Canada

## Abstract

**Background:**

Until recently, little attention has been paid to local innovation capacity as well as management practices and institutions developed by communities and other local actors based on their traditional knowledge. This paper doesn't focus on the results of scientific research into innovation systems, but rather on how local communities, in a network of supportive partnerships, draw knowledge for others, combine it with their own knowledge and then innovate in their local practices. Innovation, as discussed in this article, is the capacity of local stakeholders to play an active role in innovative knowledge creation in order to enhance local health practices and further environmental conservation. In this article, the innovative processes through which this capacity is created and reinforced will be defined as a process of "ethnomedicine capacity".

**Methods:**

The field study undertaken by the first author took place in India, in the State of Tamil Nadu, over a period of four months in 2007. The data was collected through individual interviews and focus groups and was complemented by participant observations.

**Results:**

The research highlights the innovation capacity related to ethnomedical knowledge. As seen, the integration of local and scientific knowledge is crucial to ensure the practices anchor themselves in daily practices. The networks created are clearly instrumental to enhancing the innovation capacity that allows the creation, dissemination and utilization of 'traditional' knowledge. However, these networks have evolved in very different forms and have become entities that can fit into global networks. The ways in which the social capital is enhanced at the village and network levels are thus important to understand how traditional knowledge can be used as an instrument for development and innovation.

**Conclusion:**

The case study analyzed highlights examples of innovation systems in a developmental context. They demonstrate that networks comprised of several actors from different levels can synergistically forge linkages between local knowledge and formal sciences and generate positive and negative impacts. The positive impact is the revitalization of perceived traditions while the negative impacts pertain to the transformation of these traditions into health commodities controlled by new elites, due to unequal power relations.

## Background

### Introduction

Until recently, little attention was given to "local innovation" stemming from traditional knowledge as well as management practices and institutions developed by communities and local actors. There is still a widespread tendency to regard traditional knowledge as unorganized and 'primitive' or as a treasure to store and document for posterity before it is lost, rather than seeing the dynamics that underpin the creation and dissemination of knowledge, in which local communities are key protagonists.

Innovation is still often viewed as being mainly science-led and created from the outside, and subsequently transferred to technology users, such as local communities, considered the recipients, of such innovation. This approach is a based on a clear institutional separation between innovation users and creators. Similarly, innovation is associated with activities linked to knowledge seeking and generation, that are organized separately from the activities involved with knowledge transfer and application [1]. There is thus a division of labour whereby scientific bodies are conventionally organized into a hierarchy of institutional structures with a uni-linear flow of resources and information from top to bottom [2]. In general, development and innovation is still thought as coming from the North and scientific enterprises towards the South and populations. In this paper, we analyze the initiatives of two non-governmental organizations (NGOs) in India to see how innovation rather stems from all actors implicated while being strongly influenced by current leading global networks.

Following the above considerations, this paper explores the issues linked to the participation of the community and of other local actors in the creation of knowledge through relationship building, community planning, decision-making and action within local institutions. Our focus is to understand the community participation process and the interaction that takes place between the various partners involved in the generation and dissemination of knowledge.

The key questions addressed in this paper revolve around understanding how communities and local actors build on their social and cultural traditions and practices to create and adapt their knowledge in order to favour innovation; how their capacity to learn and innovate can be recognized and facilitated to contribute to local development; and how local capacity is built to facilitate continuous learning that sustains innovation.

Understanding this dynamic is important to gain insight on the mechanisms and processes that allow the use of traditional knowledge to enhance socio-economic development. The issues studied go beyond understanding how to perform efficiently in a capitalist environment; our focus is to understand how local skills and knowledge are enhanced in order to promote innovative results that will benefit the community on a social level.

Regarding definitions, some authors [3,1] affirm that an innovation *process *consists of putting knowledge into use, whether it is new, accumulated or simply used in a creative manner in response to market opportunities or other social needs. This process is characterised by the presence of diverse agents and complex interactions between them [2]. Innovations are seen as social constructs and, as such, they reflect and result from the interplay of different actors, often with conflicting interests and objectives, and certainly with different degrees of economic, social and political power [4].

The *innovation systems *[[3,5,6], and [2]] concept provides an alternative framework to look at *innovation processes *from a systemic perspective. In the current development discourse, the innovation system concept is increasingly referred to as a metaphor to indicate the need for a much wider perspective on relevant decision-making procedures than what has been used in the past. The *innovation systems *concept can be seen as non-linear and reflexive [7]. The *innovation systems *framework "opens the 'black box' of innovation" [1] to include the roles of different innovation agents, the types and quality of interactions between them, and the formal and informal institutions that structure innovation processes [3]. The notion that innovations are the product of networks of social and economic agents interacting with each other and, because of this interaction, create new ways to deal with social or economic processes, is explicit in the innovation system concept. As [5] argue, this concept highlights the critical importance of idiosyncratic, inter-personal and interorganizational relationships and partnerships for innovation. 'Social capital', that is, the ability to form cooperation relationships, is a key ingredient of effective innovation systems.*Innovation agents *are individuals or organizations from the public or private domain who have the ability to bring about change, or have the power 'to say' and 'to act' upon the actions of others [8]. In effective innovation networks, the various partners must bring resources and capacities that are valuable to the rest and that contribute to the common goal; that is why closed networks of "poor people with poor people" are often not particularly effective in producing useful and sustainable innovations [9].

*Capacity*, like sustainability, is an elusive concept. In literature, it is described as both a process and an outcome, as dynamic and multidimensional [10]. In our analysis, we will refer to the following definition: *"Capacity strengthening is an ongoing process by which people and systems, operating within dynamic contexts, enhance their abilities to develop and implement strategies in pursuit of their objectives for increased performance in a sustainable way" *[11]. This definition seems appropriate for the approach we are pursuing since it emphasises the dynamism and sustainability elements of capacity building. In our analysis, we propose to elaborate on the notion of "ethnomedicine capacity", which refers to the capacity of enhancing traditional ethnomedicinal knowledge to open up new avenues for the socio-economical development of local populations.

In this article, we refer to healing-related practices with the terms traditional medicine (TM) or ethnomedicine. A general characteristic of these practices is their integration into daily life as well as the overall lack of documentation and standardization [12]. In this sense, we use TM or ethnomedicine to describe a very dynamic form of local knowledge. The indigenization of global health knowledge fits well into this understanding of ethnomedicine in the sense that biomedical or scientific practices are always localized [13]. Nevertheless, we will focus on what early anthropologists and, later, the WHO and most NGOs today refer to in terms of TM or ethnomedicine: in a very broad sense, all healing practices at the periphery of the global health networks, meaning they are visible to global health networks but are considered the weaker 'other'. TM can further be distinguished between 'guided' knowledge (the expert knowledge of the healers, which relies upon relations with the ancestors, the earth, among other things) and 'non-guided' knowledge (common knowledge) [14], while only the latter can be grasped by these initiatives to promote ethnomedicine. In the more classical sense of the term, ethnomedicine implies documenting and transforming parts of TM (the non guided knowledge) into measurable entities. For scientific classification, standardization and translation purposes, the term ethnomedicine has largely been narrowed down to the properties of natural *materials *used in their wild form, or part of a preparation or mixture. These materials include plant-based or "*herbal medicines"*, as well as animal parts and minerals. "Folk" traditions and other TM systems use a large number of medicinal plants. Because of the extensive use of plants, the concept of TM is more often linked to plant-based medicines. Although based on natural products, indigenous medicines are not "found" in nature but are rather products of traditional knowledge. TM includes knowledge concerning medicines and their use (appropriate dosage, how they should be administered, etc.) as well as the procedures and rituals applied by healers in their traditional healing methods. When TM encounters biomedicine, it is reduced to its measurable entities. However, when these entities maintain their link with a local culture, they become what we have called an 'ethnomedicine capacity'.

The processes through which this capacity is created and reinforced constitute innovation. As we will see, this form of innovation centred on TM involves several actors. These health traditions are dynamic, innovative and evolving, and consist of various health practices based on local epistemologies and empirical experience [15]. Since our analysis will mainly focus on ethnomedicine capacity creation and dissemination at different levels and among various stakeholders, we have decided to adopt a double approach to analyze *capacity development*: we will use an organizational *approach *and a *participatory process approach at the community level*. The *organizational approach *focuses on identifying the capacity elements or components within an organization [[16,17], and [18]]. Since the capacity development capacity is, by its very nature, multilevel, holistic and somewhat interrelated, in the sense that each system and part is linked to another, we will use a combined "closed" and "open" system perspective. Our analysis will also focus on the influences coming from the external environment since the organization is part of the network. The *participatory process approach *at the community level is based on a vision, which sees development as people-centred and non-hierarchical. Based on this approach, *capacity development *must be a participatory and empowering partnership that allows those involved to feel a high degree of ownership in order to achieve the intended results. Among individuals, networks of positive social relations are known as "social capital" [19,20]. The notion of social capital can be extended to relations among associated groups of more formal organizations, with each organization operating within the network. Such an infrastructure of relationships provides organizations with greater access to resources and helps structure relations among them [21].

We will examine the partnerships between the local communities and the Foundation for the Revitalization of Local Health Traditions (FRLHT) and the Covenant Centre for Development (CCD), two related Indian NGOs based in Bangalore (Karnataka) and Madurai (Tamil Nadu) respectively. Their aim is to revitalize Indian local health traditions through a range of field activities and research and extension programs.

## Methods

The field study took place in India, in the State of Tamil Nadu, in the districts of Dindugal and Virudhanagar Ramanad over a period of four months in 2007. The data was collected through formal individual and group interviews. To complement the data collected in the interview process, we have also used participatory observations, such as attending village organizations' meetings, observing how their members cultivated, gathered and used local medicinal plants. Our ethnographic approach is thus based on systematic observations, partly through participation and partly through various types of conventional interviews.

The first set of interviews was carried out with a sample of 15 FRLHT and CCD members, including the directors, programs officers and field officers. These in-depth interviews focused on the main characteristics and the perceived outcomes of this partnership initiative in terms of creation and dissemination of innovation stemming from TM, the contribution of the different network partners and the present and future challenges in the implementation and development of this initiative. The interviews, which consisted of open questions, lasted approximately 60 to 90 minutes.

The second set of interviews consisted of individual and group interviews with the villagers of three village organizations (*Sangha*, *Kalasam *and *MahaKalasam*) who were taking part in the FRLHT and CCD initiative. A sample of 20 members of each village organization was interviewed. In order to ensure a representative sample of the village society, parameters such as age, caste belonging and economic conditions were taken into account. The individual interviews consisted of structured and semi-structured components. The villagers were selected using a snow-ball technique in some of the CCD workers who worked in the area were asked to point us to some members of the village institutions whom we could interview. Their suggestions were based on the active involvement of these persons right from the outset of the initiatives organized by FRLHT and CCD and who, for this reason, were considered knowledgeable resources. Since the villagers to interview were pointed out by the main NGOs involved in the study, we have extended the sample in order to reduce the risk of bias. Half of the total sample was matched with a sample of randomly selected villagers who were taking part in the village organizations. While selecting interviewees, we tried to keep a balance between the socio-economic variables deemed relevant (e.g. age, caste, economic background).

Two focus groups with members of two different village organizations randomly selected were also carried out in order to complement and cross-check the data previously collected through individual interviews. In order to facilitate interaction between members, each focus group consisted of 12 members from the organizations that were not been previously interviewed.

The topics for the above-mentioned focus groups were centred on the role of local institutions in the FRLHT-CCD initiative, on the interrelations between village organisations and the other network partners. In these interviews, we also discussed the future challenges at the community level and put a particular focus on the maintenance of knowledge on traditional medicine.

We also conducted ten interviews in the villages with local folkhealers that are taking part in the initiative. This has enabled us to take into consideration local expert (or guided) knowledge on healing and hence gain some general insights into the local health system and the role played by the folkhealers in the initiative and in the conservation of traditional medicine. The folkhealers were asked to give their opinion on the importance attributed to their practices and knowledge by the villagers.

To analyze the interview transcripts, we have used content analysis. The themes that emerged from the data were grouped in categories such as capacity building, participation in the process of knowledge creation and diffusion, interrelationships with external organisations (in particular with FRLHT and CCD), degree of interest and acceptance of younger generations towards ethnomedicine and related future challenges (ecological constraints, folk healer acceptance, integration of local and scientific knowledge).

## Indian NGOs and TM

At the beginning of the 90s, FRLHT and CCD launched a joint effort aimed at conserving the TM of rural communities in the State of Tamil Nadu. This consisted in documenting TM in relation to medicinal plants and the enhancement of local capacity building through workshops and courses targeted to rural communities in Tamil Nadu. One of the initiatives recently resulted in the creation of a community-based enterprise, called the Gram Mooligai Limited Company (GMCL), which gathers medicinal plants, and produces and markets phytomedicine based on ethnomedical knowledge. It relies upon documenting this type of knowledge, enhancing local capacity-building through workshops and courses targeted to rural communities in the rural areas of Tamil Nadu, and organizing village-level commercial activities centred on traditional knowledge in ethnomedicine and local plants.

These two NGOs were selected for this case study because of their active involvement in participative projects with local communities dealing with the revitalization of local traditional health systems as described below. Very few NGOs are engaged in these issues in the regions under study.

The FRLHT was established in 1993 in Bangalore (Karnataka). FRLHT seeks to revitalize Indian local health traditions through a range of field activities and research and extensions programs.

The main areas of action are:

• the conservation of the natural resources used by traditional medicine systems;

• the demonstration of the contemporary relevance of the theory and practice linked to traditional medicine systems; and,

• the revitalization of the social processes (institutional, oral and commercial) to ensure transmission of the traditional knowledge.

CCD is a related NGO based in Madurai (Tamil Nadu); it was promoted in 1988 by a team of social work students and teachers. This NGO has gained experience in mobilizing rural communities, notably in the context of micro-credit with the creation of village organizations.

### Processes through which ethnomedicine capacity is generated

"Ethnomedicine capacity" originates from the interactions between the scientists of the FRLHT, the CCD members and the villagers, particularly the folk healers. This involves *research *and *action *and consists in the documentation, research and conservation of medicinal plants, in the dissemination of knowledge and in the increase of awareness among the villagers regarding traditional practices and uses of ethnomedicine. In an effort to document the ethnomedicinal knowledge of local communities, FRLHT and CCD pooled their expertise. As a non-governmental organization actively interacting with the village organisations through the implementation of micro-credit and community-outreach initiatives related to natural resources management, CCD has acquired extensive knowledge and the acceptance of local communities. FRLHT's expertise is focused on participative research within local communities in order to identify medicinal plants traditionally used by the villagers. It also focuses on compiling inventories of local health systems.

FRLHT and CCD's joint involvement in the documentation of medicinal plants began in 1994 with the *naattu vaidhyar*'s (traditional folk healers) information, health practices, remedies and local species of medicinal plants used to prepare cures. For the most part, local health traditions are not documented in India. According to the nomenclature database developed by FRLHT, there are about 50,000 local names for the roughly 4,800 medicinal plants used in folk medicine. It is important to underline that, in India, the orally transmitted "folk" system practiced by village physicians/folk healers and tribal communities coexist with "scientific" (*Sasthreeya*) systems such as Ayurveda, Sidha, Unani and Amchi, which are based on organized, codified and synthesized medical wisdom that have strong theoretical and conceptual foundations as well as philosophical explanations. FRLHT documented the knowledge of about 200 practitioners. Of these, about 55 were *Visha Vaidhyars *(poison healers) and 31 were traditional birth attendants. In total, 578 medicinal formulas have been recorded. In addition to the health and medicinal aspects, information on their socio-economic and cultural aspects was also collected. Besides health practitioners, approximately 600 women in 400 households from 60 villages were interviewed by FRLHT and CCD to record the household remedies associated with medicinal plants. These practitioners have participated in the documentation of more than 700 practices. FRHLT and CCD documented the pharmacopeian standards for 61 plants with the help of local folkhealers. A report was prepared on the simple formulations for Primary Health Care, which could make this catalogue useful on a global and local level.

Understanding how people use the local resources in local health practices has been essential for FRLHT and CCD. This has provided the basis for an effective methodology that must be documented in order to promote the use and conservation of these practices, maintain these traditions and also offer some "recognition" to the people withholding this knowledge. The documentation process suggests recognizing the value of this form of traditional knowledge. Although the intention is usually genuine, this could transform this knowledge to a point where it becomes unrecognizable or, as we propose here, it should rather be seen as innovation, or a re-invention of traditional knowledge which may be re-appropriated by people in their daily practices, or not. In the latter case, it may become new expert knowledge that remains within the confines of the NGOs' leaders, or within the hands of what some refer to as a new 'elite'.

Another step in the documentation process entails the standardization of plants extracts developed by FRLHT in conjunction with the folkhealers using modern techniques and models. The director of FRLHT explains: *"Traditional healers often depend on subjective parameters like taste, smell and unwritten knowledge to determine the quality of the ingredients and the efficacy of medicines. FRLHT is trying to document these parameters using modern methods in order to make them more objective and ensure anyone can check them." *In this quote we find a limitation to understanding scientific knowledge as 'objective' rather than as both subjective and objective and in this way similar to 'traditional knowledge'. This limitation has been addressed thoroughly by various anthropologists such as Bruno Latour and by the field of medical anthropology in order to understand the cultural embeddedness of biomedicine and its associated processes, including the demonstration of efficacy through randomized clinical trials [[22-24], and [15]]. In this view, both science and local knowledge need to be understood through the same ethnographic eye [25]: both in a subject-object relation, neither objective nor completely subjective, yet a mixture of both. Hence, there are no 'objective' parameters that someone can check, only shared parameters emerging from innovation. Conciliation, revising or translation of these methods would also need to comply/compromise for the innovation to be maximised and stem equally from both sets of knowledge; power/knowledge relations would need to change or be dismantled [26] for further innovation capacity to be developed. This would make the claim of FRLHT limited to the possibility of making only very partial aspects of traditional medicine visible, with the increased possibility of lost efficacy and of lack of appropriation of the 'new' knowledge by the people.

Apart from the methodology for documenting medicinal plants, FRLHT has developed, in conjunction with the CCD and the local communities, the Community Health Traditions Register (CHTR), for the protection of community Intellectual Property Rights. Participatory workshops are offered to interested communities. These workshops help communities identify and document the main medicinal plants, their economic significance to the ecological history contained in the biota on indigenous and local community lands and territories. The inspiration for this now widespread initiative arose not only from the desire to protect community intellectual property rights, it was also felt that through a documentation process, communities could also renew or develop resource management strategies promoted by FRLHT but led by the villagers. It is important here to mention that both the Federal Drug Association (FDA) and the World Health Organization (WHO) have recently accredited value to medicinal plants for their historical use. Ironically, it can only provide them with value if these are documented [15]. This explains how such initiatives like the FRLHT initiative can actually enhance 'traditional' knowledge, at least in this new form. Here again, one can argue that simply codifying knowledge is not a very effective strategy to keep this knowledge system alive and that other measures are necessary at the local level, such as preserving local ecosystems and recognizing the value of this knowledge in itself without trying to impose western development models, although the latter are inevitably also modified as they are offshored (see for example in [27]).

### Processes through which TM is diffused

Naturally, the next step of this knowledge acquisition process through documentation and research is the sharing of such knowledge with the groups immediately concerned: the local villages and their local institutions (*Kalasams and Mahakalasam*). FRLHT currently specializes in developing needs-based training courses and educational events as a way of supporting the process of preserving and revitalizing the Indian Medical Heritage. This is the process that displays the most obvious innovation, both in 'new' knowledge that is created from this encounter and documenting process and in linking knowledge to a cultural identity or struggle. This latter link between knowledge and politics in relation to indigenous knowledge, and more precisely between the promotion of traditional knowledge on plants and the dignity of indigenous people, is also evident in other projects such as The International Center on Indigenous Phytotherapy Studies (TICIPS) project in South Africa (the second author is currently conducting research with this organization).

In order to educate villagers and increase their knowledge of medicinal plants and traditional health practices, a Medicinal Plants Conservation Park (MPCP) was developed by CCD in the Madurai district on a campus named Sevayoor. The park consists of an Ethno-Medicine Forest (EMF) that spans 33 acres and boasts a collection of more than 500 plants species. This park has been conceived as an open space where humans, in this case local villagers, other communities, students, etc., can see all the medicinal plants available in that zone and learn about their various therapeutic uses. Similar regional resource centres are also being set up in Natham and Nagapattinam. These centres function as Community Conservation Centres (CCC) as well, and are owned and managed by the local communities. A *Naattu Vaidhya *Dispensary as well as a medicines preparation unit are also housed at Sevayoor, under the supervision of the *Vaidhya *residing in the campus. Two village organisations look after the dispensary and prepare medicines according to demand and sell them under their group's brand name. This is only one example of how TM has successfully been transformed into a commodity.

### Linking innovation, TM and entrepreneurial capacity: the creation of GMCL

The local communities' needs were assessed in a study preceding the Kitchen Herbal Garden (KHG) program in 1999. This evaluation was the first step that led the community and CCD to form a community-based enterprise active in the medicinal herbs sector (the GMCL referred to above). This study for the KHG program was conducted in conjunction with the local village organizations (*Kalasam*) to analyze changes in community health, food regime and routine, the ability to manage their medicinal needs, etc., and to evaluate community participation. This study showed that, in poor households, health expenses are high and that, for rural women who have very little access to resources, their own health appears last on the list of priorities and is often neglected. The GMCL involving the local villagers was started in 2001. This innovative business model is a public limited company where the majority of shares are held by medicinal plant gatherer and cultivator groups. GMCL supplies medicinal herbs to Indian pharmaceutical enterprises (such as Himalaya Drug Company, Natural Remedies, Ompharma, etc.) and plays an intermediary role between these companies and the local farmers. It also markets phytomedicines produced by local communities using their traditional knowledge. Another objective of GMCL is to promote and maintain sustainable cultivation and utilisation of medicinal plants, and to educate the villagers on the importance of using natural medicine based on plants for their basic healthcare. The training programmes organized by FRLHT and CCD helped women recognize and value this new form of local knowledge of medicinal plants. This has increased the capacity to study, document and monitor traditional knowledge on medicinal plants and their use, and spurred the inventory of medicinal plants and of the local biodiversity through biodiversity registers, among others. An increased awareness of the importance of phytomedicines as an alternative way to cure themselves and their family has also been observed. A member of a Sangha in the village of Umlallinat emphasized: *"I have associated with GMCL because I want to promote our own medicine. Natural medicine, which uses plants, does not have many side effects. Today, when we use allopathic medicines, we are inviting hundreds of other diseases while treating one disease. By marketing our products, we can give a new life to our ancient medical system and make it more popular. Since this system is a part of our national heritage, we need to respect it and promote it. Our efforts are geared in this direction. As we know, this system was developed in our villages. Hence, we should teach our villagers that by adopting this system, they can use the natural resources available in their own environment and eliminate their diseases without side effects"*. This view of TM focuses solely on positive effects and neglects important subtleties and notions, such as the fact that medicinal plants can also be poisonous if not used properly [28]. Nevertheless, it does serve to promote a new commercial form of TM that can compete with allopathic medicines to a certain extent, which are currently seen as a panacea the 'commodification' of health model [29].

Aside from this possible frail therapeutic claim - that TM has no side effects - this community enterprise currently provides livelihood opportunities to over 1,200 families, mostly landless labourers, organized in over 160 gatherer village organizations (*Sanghas*), and to another 400 families of small- and medium-sized farmers, also organized in village groups. Its trade volume has grown from 30 tons in the first year (2000) to 500 tons in the 4th year (2004), and turnover has increased from 1.5 million rupees to 9 million rupees. This may indirectly enhance the health of many villagers although new local elites monopolize much of the medicinal products trade, also through GMCL. This phenomenon is likely to be associated with the caste system as well as the capitalist system, which obviously leads to individual gain. The issue of "elite capture" regarding conservation and development projects is well-documented [30]. It is not the primary objective of this paper to examine the possible monopolization of the herbal market by outsiders, such as local traders, and this issue would deserve further investigation.

### Increase of ethnomedicine capacity at a horizontal level: the interaction between GMCL and the other village organizations

The interviews highlight that GMCL is well anchored in the *village network organizational structure*, which is composed of *Kalasams *and *Mahakalasam*. CCD previously established these village organizations in order to achieve collective savings that would be forwarded as credits to members of the group in times of need. They are structured at different levels, like the *Kalasam *and the *Mahakalasam*. Two thirds of the villagers interviewed reported that the participation of *Kalasams *and *Mahakalasam *goes beyond commercializing GMCL products; they also involve a broader commitment from an earlier stage. These villagers emphasized how the pre-existence of village organizations has allowed GMCL and members of the *Sanghas *to benefit from different village centered services already available in the village, such as credit, training, etc. The *Mahakalasam *in Pulvakkarai, Suranam and Natham villages act as head-offices for the BDS (Business Development Service Center) and the CFC (Common Facility Center). The BDS and CFC were created with the collaboration of CCD in order to support the various livelihood activities in the area and promote entrepreneurship among women. Currently, BDS provides services to about 1000 medicinal plant gatherers and cultivators. The centers offer a myriad of services, such as capacity building programs, entrepreneurial training, inter-mediation with financial institutions, effecting linkages with service providers and markets, value-addition services, tool kits and other equipment, alternate building materials, documentation services for their practices, business information services, assistance with sales outlets, and guide and manual development. The extent to which the social stratification in the villages, because of caste belonging and of the socio-economic background of the villages, may hinder full access to the services provided to various members of the local organizations still needs to be understood. This could in turn accentuate social discrimination and, in the long term, undermine the effective participation of all members of the community.

## TM and Innovation

In the above sections, we have identified some of the key features that emerge in the case studies' objectives, in terms of promoting successful innovation processes and enhancing the innovation capacity related to traditional knowledge. In this section, we will further analyze their success and limits.

### 1. Processes that integrate local and scientific knowledge

One of the important aspects highlighted in the case studies is the importance of seeing local and scientific knowledge as a coproduction. Local knowledge associated with the use and conservation of medicinal plants is either codified in ancient scriptures or folk-based and transmitted from one generation to the next in the form of community-based health traditions. The integration of ethnomedical knowledge and scientific know-how have allowed the communities in Karnataka and Tamil Nadu to successfully enhance their ethnomedicine capacity and promote their own form of development, at least to a larger extent than more 'traditional' forms of top-down development. However, new forms of TM is found to result from the process, such as local 'elite capture'. Clearly, the idea of ethnomedicine and the reality is a definite essential asset of the innovation process; the limits of this participation are the 'externally driven' standardization and codification processes required for the plants to be recognized, which have the effect of disbanding the knowledge from the protocols and epistemologies in which they were previously embedded.

### 2. Effective partnerships and network structure

One of the interesting aspects of these case studies is the way this programme has developed a variety of partnerships and networks externally and at the village level, creating new 'global assemblages' in some way, to borrow a concept introduced by [31]. These have occurred in a variety of ways and across different types of stakeholder groups. For example, in the field, village community organizations such as *Kalasams*, *Mahakalasams *and *Sanghas*, NGOs and training centres now have extensive cross-linkages that are both formal and informal. Because these are now extensive, a route has been paved to develop new forms of knowledge based on plants.

One of the strengths of the approach taken by network stakeholders to promote ethnomedicine capacity is the focus on local priorities and needs. These priorities and needs have been identified and analyzed through participative assessment exercises as well as in the field studies mentioned. The action research undertaken consisted in an iterative inquiry process that balances problem-solving actions implemented in a collaborative context with data-driven research in order to understand the underlying causes of future changes [32]. However, the establishment of sounder partnerships with outside organizations could still be enhanced to enlarge the scope of the networks. Networking with other governmental and non-governmental organisations, and heightening mainstream institutions' awareness, such as universities and policy makers, is another important line of action that could be strengthened in the future. This promotional strategy may provide the basis for a national health system that supports local health traditions, with the ultimate goal to ensure effective and affordable care for all who need it.

### 3. Capacity-building and human capital development

The training organized by FRLHT and CCD at the village level underpins the creation and dissemination of ethnomedicine capacity as well as its enhancement. During certain events, such as local healers' conventions and village botanists' workshops, local village communities learned new skills regarding identification of medicinal plants, herbarium preparation and new uses of medicinal plants. This has enabled them to recognize and value local knowledge relevant to medicinal plants' use and conservation. In the end, this creates a shift from a form of *expert *knowledge, mainly possessed by the folk healers, towards a form of common knowledge, more widespread at the community level. Capacity building rooted in the recognition of community capacities and institutions can increase the emphasis on partnerships with other stakeholders. This aspect is particularly important in order to increase the effectiveness of the role of the community in using traditional knowledge and local resources for their own benefit and to enhance the sustainability of community initiatives in this regards. However, the enhancement of the ethnomedicine capacity through a process of capacity building does not necessarily *protect *traditional knowledge from the risk of misappropriation from outsiders as it rather now relies on inside experts trained to assess and evaluate their resources and generate biological databases that exceed the communities' capacities.

### 4. Social capital and collective action underpin knowledge sharing, learning and collective action

Organizational theory and empirical evidence support the notion that knowledge is socially constructed. A process of mobilization and collective action develops a shared cognitive system and shared memories. These forms of organizational cognition, which call for the understanding of events, open the opportunity for social interpretation as well as the development of relatively dense interpersonal networks for sharing and evaluating the information, thus creating effective learning systems [33]. In the approach followed by FRLHT and CCD, there is a clear focus upon understanding the experiences of the villagers in their everyday life; there is an equally strong focus on rendering those experiences collective by taking part in the same activities related to TM knowledge. This element - sharing knowledge on the uses associated with medical plants - has been emphasized by one third of the participants. In this respect, a middle-aged woman belonging to a *Sangha *stated:*"I am a member of this Sangha. This gave me the possibility to meet other women and talk about the medicinal plants we gather and use on daily basis for our family and our children. This is very important since we can exchange information among us and learn more of the medicinal uses of the different plants, especially from the older women. " *Although this aspect is particularly important, the interviews also show that some members, particularly the young ones, have trouble actively participating to this process of knowledge sharing with the older members. The statement of a young woman illustrates this:*"Since I have been a member of this Sangha, I have learned a great deal about medicinal plants and their importance by talking with other women of this association. However, some older women sometimes protect their knowledge and do not share it with all the members on equal basis"*. This quote relates to *expert *knowledge acquired by older women and these are usually as accessible as the knowledge acquired by a botanist; only what (s)he is able to explain can be shared, never all the acquired skills and experience. Folk healers are nevertheless usually protective of their knowledge and this should be considered as very different knowledge than the new commodified, common and non-guided shared knowledge that these Indian NGOs are creating.

The presence of these grass-roots organizations allows an increased sharing in basic neo-traditional knowledge among their members but also constitutes a specific way of coordinating personal and interpersonal relationships. This in turn helps build trust and possibly prevents opportunistic behaviours. A woman from Minitankulam village affirms: *"It is good to be a member of the Sangha. This has helped me meet other women of the village. We share our problems and we support each other. If someone is in need, or if I am in need, I know that I can rely on them and they know that they can rely on me. It is mutual"*. Despite this positive aspect, four members of the village organization, mainly middle-aged women, highlight that participation in the *Sanghas *and the social pressure that their members sometimes inflict could prevent them from taking the path they wish to follow. One of these women stated:*"My husband and I mainly work as labourers for the local landowner. We also have a plot of 2 acres that we cultivate. I was not interested in taking part in the Sangha. Many women that I know are involved. When I told them that I did not want to be involved, they became quite hostile and unfriendly. At the end, I decided to become a member in order to avoid deceiving them and to be accepted by them"*. This aspect emphasises how the establishment of these community networks sometimes create undesired external influences in the lives of the members of these village organizations.

### Challenges ahead

The case studies show that the type of development intervention promoted by FRLHT and CCD is partly successful in terms of capacity development. In order to provide a balanced picture, it is still worth noting some issues and some problem areas.

Firstly, in order to preserve TM, it is necessary to promote the local resources on which they are based. Ecological constraints pose serious challenges to the future development of programmes such as the ones deployed by FRLHT and CCD since there is no evidence that the gatherers use sustainable harvesting techniques and are fully aware of the importance of conserving medicinal plants in the wild. The continuous exploitation of several medicinal plant species from the wild [34] and substantial loss of their habitats during the past 15 years [35] have resulted in population decline of many high value medicinal plant species over the years in many areas of India [34,36]. Since the demand for medicinal plants derives from the demand for herbal medicines and related products, and the majority of Ayurvedic raw materials consist of medicinal plants, the growth of the Ayurvedic industry will consequently result in a higher demand for medicinal plants. This will put an additional pressure on the medicinal plant sector to provide specific quality raw material for the growing industry. Many studies have already addressed the problem of the economic scarcity of medicinal plants in relation to the demand arising from pharmacies in India [37]. Urgent conservation efforts are thus necessary both at the local and national levels. One possible solution might be the cultivation of medicinal plants that have become rare in order to maintain their availability in the long run. However, this possible solution requires specific propagation techniques that are not always easily available on the ground. There is also the problem of land tenure and availability, as most of the local farmers possess a limited land area. Moreover, such conservation techniques must be aligned with the sociocultural context in order to avoid imposing a western conservation approach that is far from local realities and has not been proven successful up to now.

Based on interviews with folk healers, another problem that stands out is the progressive reduction of expert TM knowledge and of faith in its efficacy, especially among younger generations. Interviews emphasised that there are important knowledge differences between generations. Almost all the elders and folk healers interviewed mentioned how youth seem to have very little confidence in traditional medicine. The main issue seems to be that a process of cultural domination is in action: the younger generations are more oriented towards western cultural models and adhesion to allopathic medicine is one of its expressions. Besides the known reasons for the erosion of cultural diversity, such as the western model of education, it cannot be denied that the establishment of western medicine and the propaganda to the effect that it constitutes the most effective form of healing therapy is very widespread among many local communities and has undermined traditional practices, on top of undermining their limits and potential side effects. A member of a Sangha in the village of Umlallinat indicated: *"people are happy with the allopathic medicines. There are two things here: the lack of awareness that medicinal plants can offer a cure, however slow and that allopathic medicine, though it cures fast, can cause lots of side effects because of its chemical composition. But people accept the latter because it is well advertised, and lot of money goes in to promoting it. The Government also promotes it. Therefore, people think that allopathic medicine is best*..."

Presently, the chances of economical survival as a healer are diminishing. The primary occupation of seven healers out of the ten interviewed is agriculture, livestock rearing or labourer. One possible way to increase the popularity of TM among the villagers is to foster the active participation of folk healers in the process. The folk healers were involved in the documentation of traditional knowledge at the beginning of the conservation programmes. Their role afterwards has been marginal since the new knowledge acquired turns towards the molecular biology of the plants to decipher the 'truth' about their efficacy and focuses on commercialisation; this in turn disregards any criteria of efficacy that the healer applies to make his healing techniques work. The disregard of the healers was especially clear after the creation of the community enterprise GMCL. FRLHT has developed a partnership with folk healers during these last few years. This partnership could be further expanded by trying to better understand the elements and sets of relations necessary for the healer to pursue his work [38]. Perhaps, the healers should be the ones training FRLHT personnel and others who would like to understand not only how to heal with plants but also the specific skills required and how the plants must be manipulated in order for them to be efficient. In order to promote TM knowledge in the long term, it may be necessary to increase the villagers' awareness of the importance of using medicinal plants as an effective and side effect free form of treatment. It nevertheless sends a somewhat 'false' message since medicinal plants also have side effects if one does not know how to use them properly. Presenting TM as a panacea may create the same problem as what we are currently seeing with allopathic medicine. A member of a *Sangha *from the Minitankulam village affirms: "*Because the villagers don't know about side effects, they are happy with the allopathic medicine... they say if you take allopathic medicines diseases are cured in one day... but what will happen next?"*. That TM has no side effects seems to be a strong message spread by the NGOs and this indicates a serious loss of the subtleties and relations that should be maintained to ensure the effective use of any medicine, whether plant-based or chemically-constituted.

If folk healers expressed a positive opinion on the GMCL medicines, this would facilitate building trust towards the GMCL products among villagers and would enhance their acceptance, perhaps in conjunction with allopathic medicines. The case of the Maddur village is emblematic. In this village, Narasimaiah, a local folk healer has played an important role in promoting the use of medicinal herbs. He affirms:*"When patients come to me (and, in general, I have known them for many months), I give them medicine for about three days. They then realize the importance of the medicine and come back for more... People usually go for English drugs or injections in emergency situations. When people come to me and treat them successfully, they continue to have faith in me. I think herbal medicine has a good future"*. The village organizations such as *Sanghas *or *Kalasams *can become key players in promoting a community based health system and in raising the villagers' awareness regarding the importance of traditional medicine, as long as they inquire further into the depths of indigenous knowledge and facilitate its pursuit through other parallel means beyond simply commodifying health based on plants for commercial purposes. New societal space needs to be created for healers to regain credibility and continue to develop expert innovative and creative practices.

## Conclusion

The case studies highlight that despite some limitations and challenges that remain regarding the conservation of biodiversity and the different perceptions of traditional medicines from one generation to another, communities and local actors effectively build on their social and cultural practices to create innovative processes centred on traditional knowledge. The efforts of FRLHT and CCD in Southern India have revitalized folk knowledge and promoted new forms of folk knowledge associated to medicine. They have also integrated local and scientific knowledge through a grassroots community groups approach. As the case studies point out, development activities that work with and within traditional knowledge and organizational structures have several important advantages over projects that operate outside them. Traditional knowledge provides the basis for grassroots decision-making, much of which takes place at the community level through village organisations where problems are identified and solutions are determined at the local level. The creation of a network of several local actors and the enhancement of the village capacity building through village organizations are important instruments to enhance the innovation capacity of the organizations.

The case studies analyzed highlight the need to link local communities to other social and economic agents whose capabilities are necessary for many substantive innovation processes to take place. The case studies also point towards a need for enhancing larger global networks with conservation policies and networks of healers, for example, who should be consulted as experts and whose work should be facilitated. These community initiatives are sustained through innovative policies and actions that entice change and innovation; the latter may have positive effects such as to revitalize or reinvent perceived traditions and essential links between plants and ethnicity, feed into new scientific knowledge and create economic opportunities. Due to unequal power relations, innovation may also have negative effects, such as the transformation of these traditions into knowledge that is unrecognizable and difficult to integrate in local practices. Innovation may also lead to the transformation of these healing traditions into health commodities controlled by new elites.

## Competing interests

The authors declare that they have no competing interests.

## Authors' contributions

The authors have made substantive intellectual contributions to this study in data collection, preparation of the manuscript and proof reading.
